# A novel mouse model carrying a human cytoplasmic dynein mutation shows motor behavior deficits consistent with Charcot-Marie-Tooth type 2O disease

**DOI:** 10.1038/s41598-018-20081-1

**Published:** 2018-01-29

**Authors:** Thywill T. Sabblah, Swaran Nandini, Aaron P. Ledray, Julio Pasos, Jami L. Conley Calderon, Rachal Love, Linda E. King, Stephen J. King

**Affiliations:** Burnett School of Biomedical Sciences, College of Medicine, University of Central Florida, Orlando, FL 32827 USA

## Abstract

Charcot-Marie-Tooth disease (CMT) is a peripheral neuromuscular disorder in which axonal degeneration causes progressive loss of motor and sensory nerve function. The loss of motor nerve function leads to distal muscle weakness and atrophy, resulting in gait problems and difficulties with walking, running, and balance. A mutation in the cytoplasmic dynein heavy chain (DHC) gene was discovered to cause an autosomal dominant form of the disease designated Charcot-Marie-Tooth type 2 O disease (CMT2O) in 2011. The mutation is a single amino acid change of histidine into arginine at amino acid 306 (H306R) in DHC. In order to understand the onset and progression of CMT2, we generated a knock-in mouse carrying the corresponding CMT2O mutation (H304R/+). We examined H304R/+ mouse cohorts in a 12-month longitudinal study of grip strength, tail suspension, and rotarod assays. H304R/+ mice displayed distal muscle weakness and loss of motor coordination phenotypes consistent with those of individuals with CMT2. Analysis of the gastrocnemius of H304R/+ male mice showed prominent defects in neuromuscular junction (NMJ) morphology including reduced size, branching, and complexity. Based on these results, the H304R/+ mouse will be an important model for uncovering functions of dynein in complex organisms, especially related to CMT onset and progression.

## Introduction

Charcot Marie Tooth disease (CMT) is an inherited peripheral neuromuscular disorder in which length dependent axonal degeneration results in progressive loss of motor and sensory nerve function. Loss of motor nerve function leads to weakness and atrophy of muscle tissue in the distal limbs. The phenotype usually initiates with excessive foot arching (*pes cavus*), which leads to structural deformity of the foot. This is typically followed by wasting of the gastrocnemius muscle, which results in gait problems such as high ‘stepping’ and difficulties with walking, running, and balance. Loss of sensory neuron function may lead to tingling and burning sensations, decreased sense of touch, defects in proprioception, hearing loss, deafness, and blindness^1^. Chronic pain and limb weakness, progressive inability to walk, balance problems, orthopedic problems, and progressive inability to use hands effectively are commonly associated complications of CMT that lead to a significant reduction in the quality of life of those diagnosed with this disease^2–5^. Worldwide, CMT affects approximately 1 in every 2,500 individuals, or about 2.8 million people. There has been no scientific assessment of the economic burden of this disease upon our society but based on the number of people afflicted in the US alone (over 125,000), this is a significant health problem for our society to face. There is currently no cure for CMT onset or progression.

There are two main underlying disease pathologies of CMT that alter normal peripheral neuronal function: deficiencies that affect myelination and deficiencies that affect axon function. Myelination pathology is further divided into four categories of disease (types CMT1, CMT3, CMT4, and CMTX) that are caused by autosomal mutations in any of at least 40 genes that play different roles in the myelination process^6,7^. In contrast, CMT type 2 results from mutations in any of a group of almost 20 genes that have the common feature of encoding for proteins that have some sort of role in axon function^8^. CMT2 variations of CMT disease do not have defects in nerve conductance velocity as seen in the other disease categories. All five categories of CMT disease have different mechanisms of onset, progression, and severity but result in very similar phenotypes^9^. Although there are now over 80 genes that have been linked to CMT, one of the largest gaps in our understanding is a direct determination of the mechanism by which specific mutations result in the onset and progression of CMT disease.

In 2011, a mutation in the Dync1h1 cytoplasmic dynein heavy chain (DHC) gene was discovered to be the cause of CMT2 disease in four generations of an afflicted family and was designated Charcot Marie Tooth type 2 O disease (CMT2O)^10^. A single amino acid change of histidine at amino acid 306 into arginine (H306R) of the Dync1h1 gene resulted in an autosomal dominant form of CMT2 that included phenotypes such as *pes cavus*, abnormal gait, lower limb weakness and wasting, learning difficulties, and reduced sensory reception^10^. A second study^11^ also identified H306R as a causative mutation in an Asian family with a similar neuropathy: spinal muscular atrophy with lower extremity predominance. Cytoplasmic dynein (hereafter called dynein) is a multi-subunit microtubule motor that transports cargos toward the minus ends of microtubules; dynein is the retrograde motor for axonal transport. The dynein motor complex is absolutely essential for nerve axonal function; dynein transports cargos including mitochondria, neurotransmitter vesicles, the microtubule tracks, neurofilaments, anti-apoptotic survival factors and a host of other axonal components^12^. Complete loss of cytoplasmic dynein function abolishes retrograde microtubule-based cargo transport in cells and is lethal in the early development of differentiated multicellular organisms^13–16^.

The identification of a Dync1h1 mutation causing Charcot Marie Tooth type 2 O provides a unique opportunity to directly study the mechanism by which a specific mutation results in the onset and progression of CMT type 2 disease. Much is already known about the structure and function of cytoplasmic dynein. Each dynein motor is assembled into a complex structure that includes a DHC dimer and various dynein intermediate chain, dynein light intermediate and dynein light chain subunits^17^. Dynein motors hydrolyze ATP to take successive steps along microtubule tracks inside cells. The DHC contains the binding sites for ATP and microtubules and provides the actual force generation of the motor complex. Dynein moves cargos at a velocity of approximately one micrometer per second, which means that in the longest axons of the human body it would take the motor around ten days of non-stop function to move a cargo from the tip of a toe to the spinal cord in a six-foot tall person. Studies have shown that separate dynein mutations can have significantly different cellular phenotypes^18,19^. It is important to note that the loss of one of the two copies of dynein heavy chain gene has no apparent phenotype in cells or in mice^13^. Therefore, the heterozygous mutations in dynein that cause CMT2 disease or other neurological disorders do not simply abolish function but must alter dynein function in some way.

To begin to understand the onset and progression of axonal CMT, we have generated and initially characterized a knock-in mouse carrying the corresponding CMT2O mutation at the same location in the Dync1h1 gene (H304R/+). Although three independent mouse lines carrying dynein mutations have been previously identified^20–22^, the mutations in those lines *Loa/*+ *(F580Y), Cra/*+ *(Y1055C)* and *Swl/*+ *([GIVT]1040[A])* do not correspond to human disease alleles and there are differences in the phenotypes of the H304R/+ mouse compared to the *Loa/*+, *Cra/*+, and *Swl/*+ mice. Examination of the H304R/+ mouse shows that it exhibits age-related neuropathic disease and locomotor defects consistent with CMT. We strongly believe that the H304R/+ mouse is an ideal model for determination of the mechanism by which the Dync1h1 mutation results in the onset and progression of CMT2O disease. Characterization of the CMT2O H304R/+ mouse model at the behavioral, tissue, cellular, and molecular level will significantly increase our understanding of both CMT specifically and the role of dynein function in neurological disease in general.

## Results

**Figure 1:**
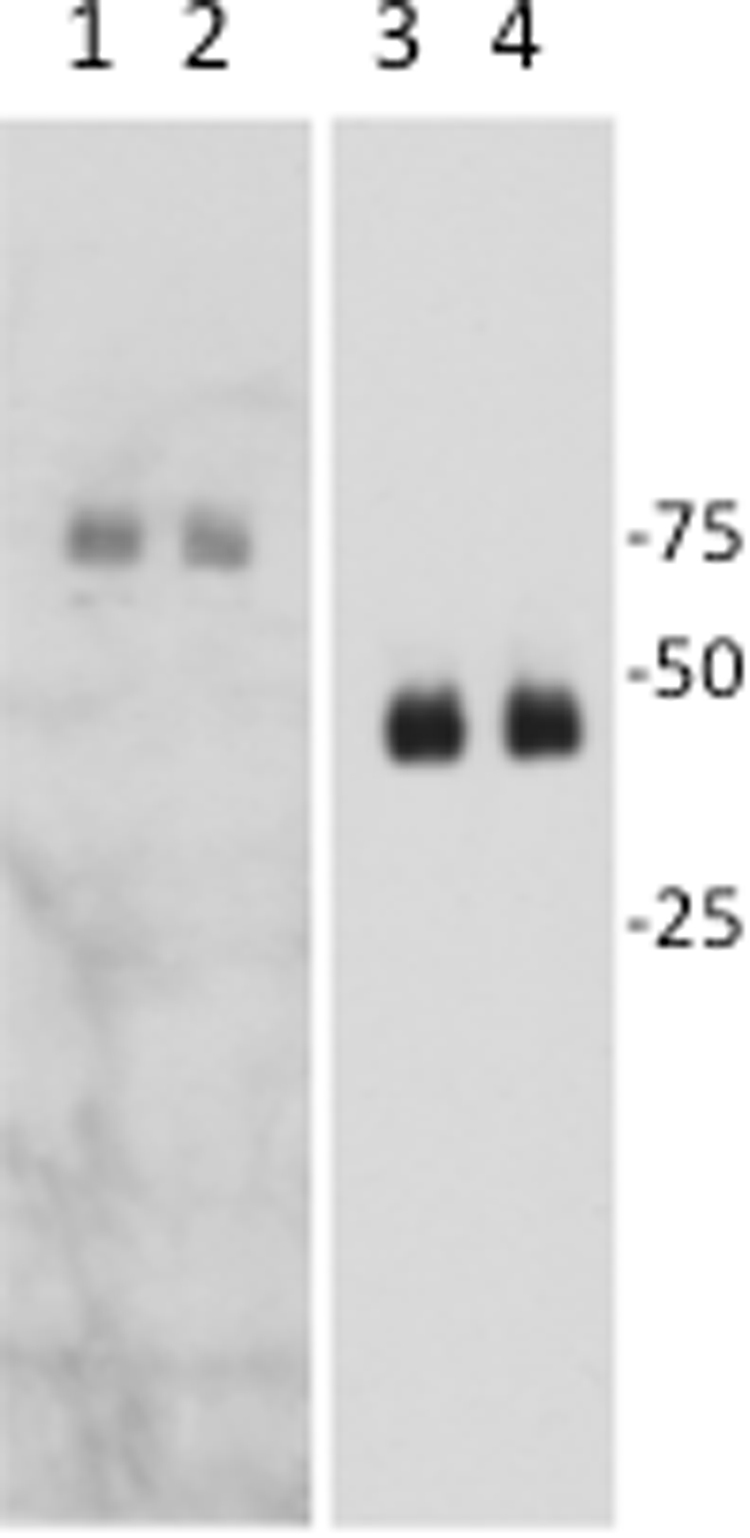
Dynein complex protein levels in wild type and H304R/+ brain tissue. Western blots of brain tissue high speed supernatant from wild-type (lanes 1 and 3) and H304R/+ (lanes 2 and 4) mice were probed for the dynein intermediate chain (lanes 1 and 2) or glyceraldehyde-3-phosphate dehydrogenase (lanes 3 and 4, loading control). The data were statistically compared between wild-type and H304R/+ using the Students *t-*test (two-tailed distribution, *p* = 0.23).

We generated a heterozygous knock-in mouse line that carries the human H306R mutation in the mouse Dync1h1 cytoplasmic dynein gene at the corresponding position (H304R). We characterized potential loco-motor phenotypes by examining both male and female littermate wild-type and H304R/+ mice in a 12-month longitudinal study. Over the time period, H304R/+ mice had no obvious physical or cage behavioral phenotypes that could be distinguished by eye from littermate wild-type animals. We tracked the general health of those animals and saw no obvious health differences in the animals outside of a modest weight gain in 3-month old female H304R/+ mice that disappeared as the mice aged. To determine the impact of the mutation on cytoplasmic dynein protein levels in mouse tissue, we performed a western blot analysis (Fig. 1). We determined that H304R/+ samples contained 97.7 ± 9.8 (mean ± S.D.) percent of wild-type dynein protein levels, a difference that is not statistically significant (*p* = 0.23).

### Loco-motor characterization of the H304R/+ mouse strain

Human patients with CMT2O disease present with a range of symptoms affecting their gait and mobility^10^. We performed standard mouse loco-motor tests on the H304R/+ mice and wild-type controls to determine if they had phenotypes consistent with CMT2 and/or those exhibited by the three previously characterized dynein heavy chain mutant *Loa/*+, *Cra/*+, and *Swl/* +  mice^20,22,23^.

#### H304R/+ mutant mice show an altered tail suspension reflex

**Figure 2:**
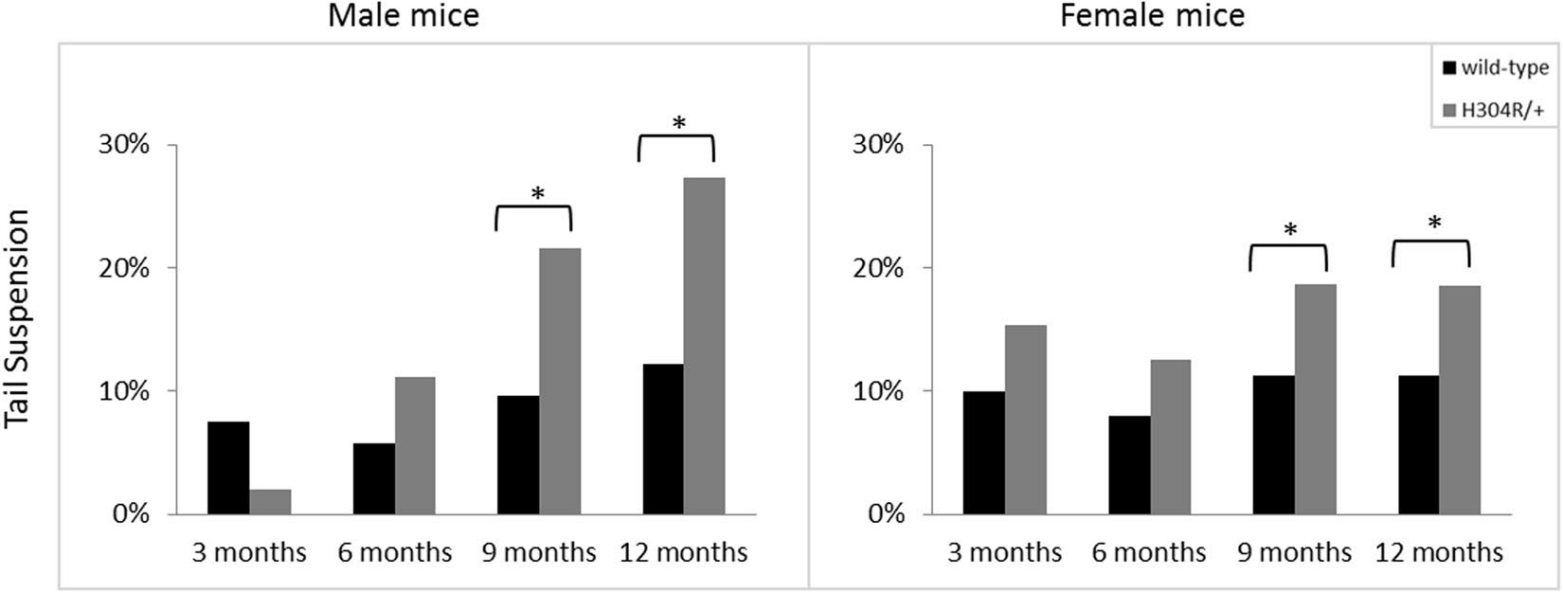
Tail suspension phenotypes in wild type and H304R/+ mutant mice. The atypical tail suspension reflex was scored and counted for each test mouse per 3-month long time intervals. The scores were statistically compared between the wild-type and H304R/+ mice using Fisher’s Exact Test (two-tailed distribution, *p* < 0.05).

Wild-type mice generally display a characteristic tail suspension reflex of splayed hind limbs held away from their body when suspended by their tails. Several studies of *Loa/*+, *Cra/*+ and *Swl/*+ mice pointed out that those mice display an atypical phenotype of clenching their hind limbs when suspended by their tails^20,22,23^. We performed a similar tail suspension assay at successive 3-month time points and found that the majority of male and female wild-type and H304R/+ mice displayed the normal splayed tail suspension reflex phenotype at each time. However, both male and female H304R/+ mice showed a significant increase (*p* < 0.05) in atypical tail suspension responses relative to wild-type at 9 and 12 months (Fig. 2, Supp. Table 1). These data suggest that the H304R/+ mice have a hind limb defect but that the defect is less pronounced than the phenotypes in Loa/+, Cra/+, and Swl/+ mice as only a subset of H304R/+ mice display the atypical response.

#### H304R/+ mutant mice have reduced muscular strength

**Figure 3:**
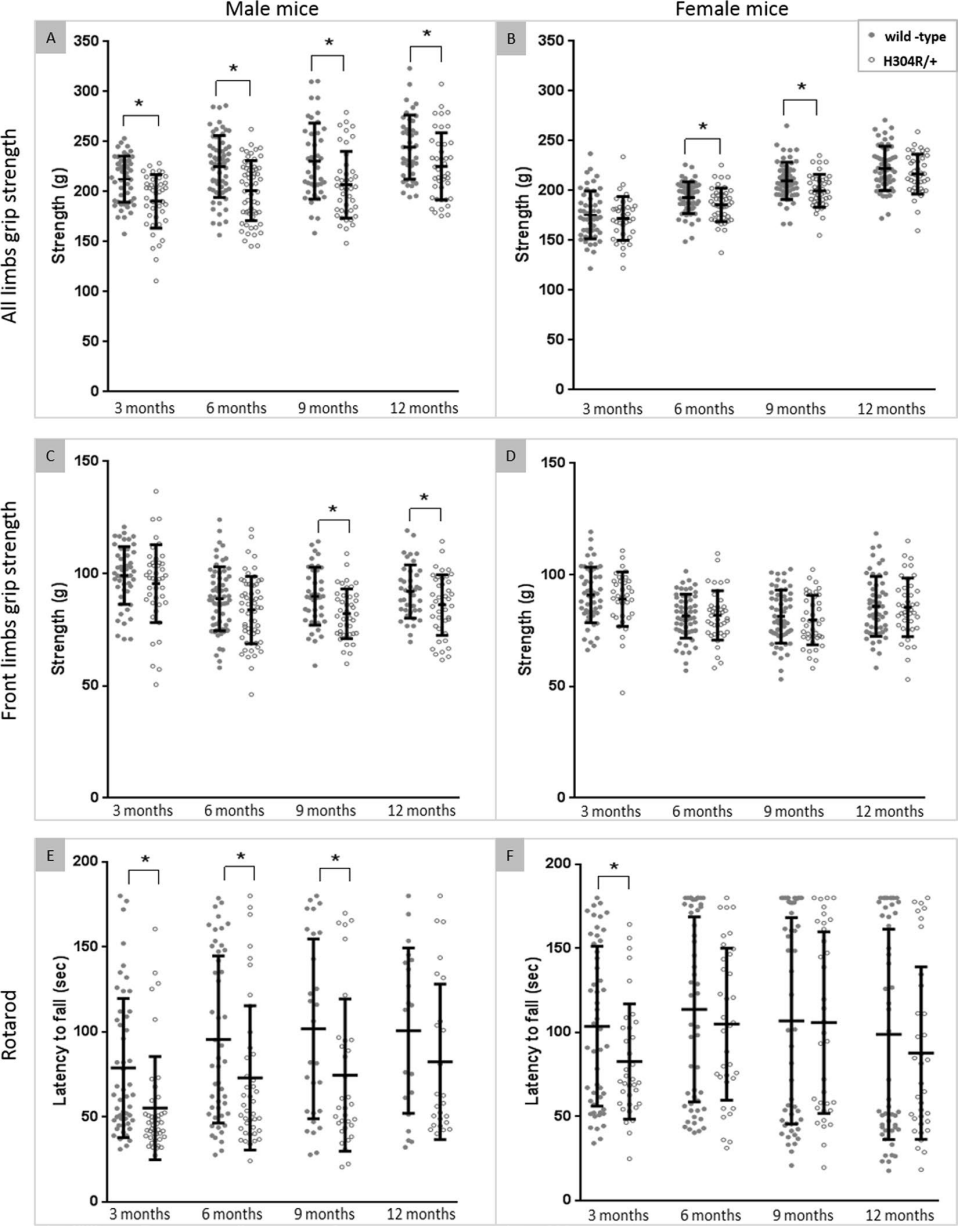
Loco-motor behavior analyses in wild type and H304R/+ mutant mice. The data show the all limb grip strength for male (**A**) and female (**B**) mice as well as the front limbs grip strength of the male (**C**) and female **(D**) mice. The rotarod performance data of male (**E**) and female (**F**) mice is shown. Each dot gives the average grip strength or rotarod value from each test mouse averaged per 3-month long time intervals. (wild-type = filled circles, H304R/+ open circles). The mean and standard deviation of each data set is shown. The data was statistically compared between wild-type and H304R/+ mice using the Students *t-*test (two-tailed distribution, *p < 0.05).

We next analyzed the limb muscular strength in our mice using a standard grip strength assay. Male H304R/+ mice exhibited significant weakness in all limb grip strength relative to wild-type male mice at all ages examined (Fig. 3, Supp. Table 2; p < 0.05). To further tease apart the contribution of front and hind limbs to this phenotype, we examined the strength of the front limbs solely. We found that there was a significant, progressive weakness in front limb grip strength of male H304R/+ mice relative to wild-type mice that occurred in the later 9 and 12-month time points (p < 0.05). These data suggest that the mice have reduced function in the hind limbs at an early age, and that front limb weakness progresses as the mice age. When we examined female mice, we found subtle defects in the combined grip strength at 6 and 9 months of age for H304R/+ females, but no defect in front limb grip strength at any age (Fig. 3, Supp. Table 3). The grip strength data is consistent with our tail suspension data, showing that there is a general trend of reduced neuromuscular function in H304R/+ animals as they age.

#### H304R/+ mutant mice have motor coordination defects

The rotarod test is a performance-based test for the evaluation of muscular endurance, motor coordination and balance in mice. We utilized an accelerating profile where the mice were tested for their ability to adjust to increasing revolutions during a ramp time followed by a consistent speed for the remainder of the assay. When we examined the ability of wild-type and H304R/+ littermates to perform this assay, we found that individual animals of a particular genotype could exhibit a wide range of abilities. However, as a group, H304R/+ males had reduced rotarod performance at 3, 6, and 9-month time points (Fig. 3, Supp. Table 2). Female H304R/+ mice only displayed a significant difference in rotarod performance at the earliest time point examined (Fig. 3, Supp. Table 3). This difference disappeared as the female mice aged. *Loa/*+ mice had been previously characterized with the rotarod test and showed similar defects^20,24,25^.

### Neuropathological characterization of the H304R/+ mouse strain

**Figure 4:**
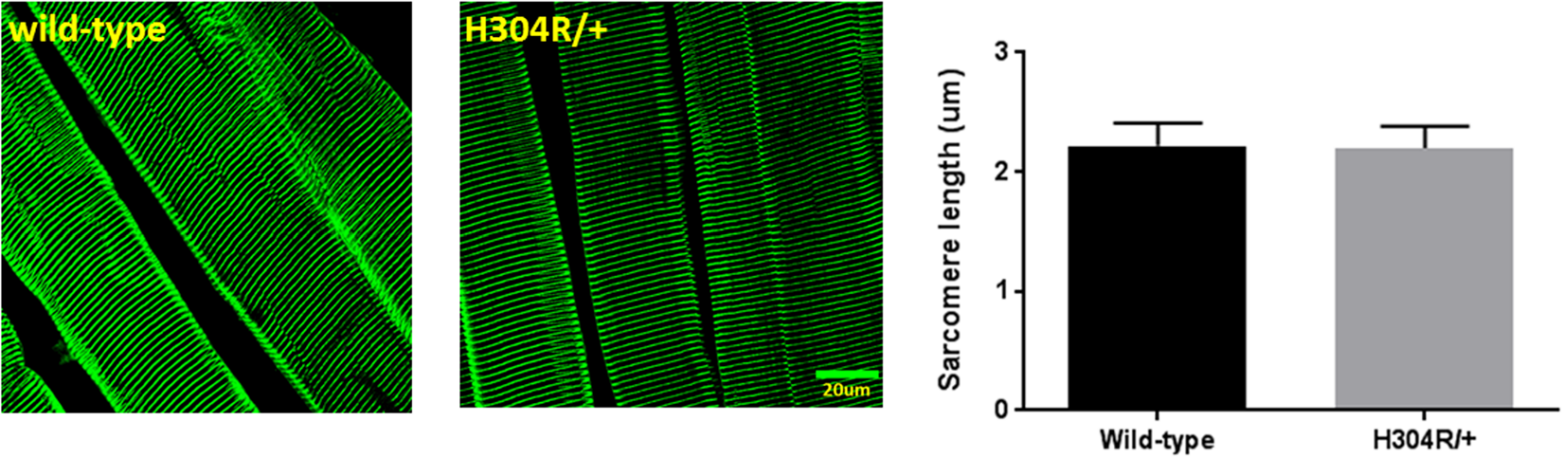
Sarcomere organization in wild type and H304R/+ mutant mice. Gastrocnemius muscles were stained with α-actinin to show the Z-lines of the sarcomeres of wild-type (left panel) and mutant H304R/+ (middle panel) mice. The right panel shows mean sarcomere length ± standard deviation of wild-type (2.22 µm ± 0.19 µm from 11 muscle fibers and 689 individual sarcomeres) and H304R/+ (2.20 µm ± 0.18 µm from 11 muscle fibers and 702 individual sarcomeres) mice. The difference between wild-type and H304R/+ sarcomere lengths was not significantly different (*p* = 0.08, Students *t-*test, two-tailed distribution).

The results of our initial loco-motor studies, altered behavior in tail suspension, grip strength, and rotarod assays, suggested to us that there might be a physical defect in the neuromuscular system in the mutant H304R/+ mice. To examine this possibility directly, we characterized the sarcomere and neuromuscular organization of the gastrocnemius lower limb muscle from male wild-type and mutant mice. We chose to examine specifically male mice, as they exhibited more significant motor behavior skills defects than female mice. Fixed gastrocnemius muscle tissue slices were labeled with antibodies for key components of those structures and imaged using standard confocal microscopy. We examined sarcomere organization by staining the Z-line with alpha-actinin (Fig. 4). We saw no qualitative differences in the sarcomere patterns between wild-type and H304R/+ mutant mice. We quantitatively measured the mean sarcomere length in the muscles and saw no significant difference (*p* = 0.08) between sarcomere lengths of wild-type and H304R/+ muscles (Fig. 4).

### Altered neuromuscular junction morphology in H304R/+ animals

We labeled 9 and 12-month tissue slices with fluorescent alpha-bungarotoxin to examine the NMJs of wild-type and H304R/+ mice. NMJs from wild-type mice displayed the classic pretzel shaped morphology with a highly branched complex appearance^26^. However, NMJs from H304R/+ animals presented a mixture of NMJ morphologies ranging from the classic morphology to those with obvious defects in size, branches, and complexity.

**Figure 5:**
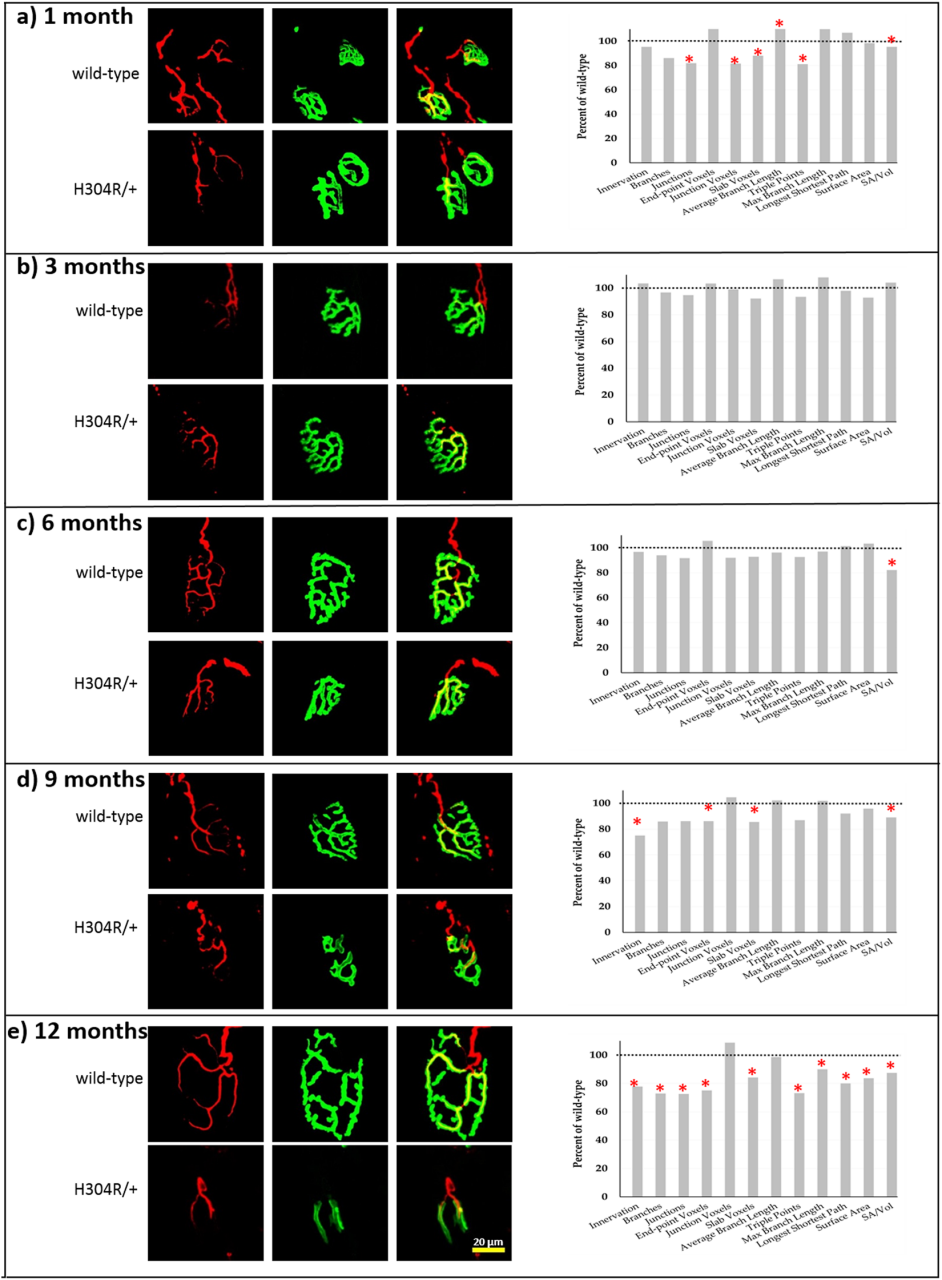
Altered neuromuscular junction architecture in gastrocnemius muscles of H304R/+ mutant mice. Confocal images of representative NMJs stained with neurofilament (red) and alpha-bungarotoxin (green) at different time points. (**a**) 1 month, (**b**) 3 months, (**c**) 6 months, (**d**) 9 months, (**e**) 12 months. Graphs show twelve parameters that were examined for the NMJ architecture in wild-type and H304R/+ animals, displayed as the H304R/+ percentage of the wild type value. Statistically significant differences between wild type and H304R/+ values are indicated by * (Students *t*-test two-tailed distribution, p < 0.05). Scale bar = 20 µm.

To more thoroughly characterize the NMJ defect, gastrocnemius tissue samples were analyzed from both wild-type and mutant H304R/+ mice from 1, 3, 6, 9, and 12-month old male animals (Fig. 5). Qualitatively, we saw abnormal NMJ complexity/morphology in 1 month H304R/+ tissue and no discernable phenotype in 3 month H304R/+ tissue. This was followed by an increasing percentage of NMJs with more obvious defective morphologies in the 6, 9, and 12-month time-points. However, not all NMJs were abnormal in the H304R/+ mice: there was a range of NMJ morphologies present at all time points. We found that an individual mouse could display a mixture of normal and abnormal NMJ morphologies and that there was a range of abnormalities present in the NMJs.

To quantitatively characterize the defects in NMJ morphology we processed image stacks of AChR fluorescence as described in Materials and Methods. Our analysis identified several interesting features that occur as the mice reach and pass through adulthood. At one month of age, H304R/+ NMJs have statistically significant defects in six of the parameters examined (Fig. 5a, Table 1). Notably, the one-month H304R/+ NMJs have fewer branches as seen by the branch, junction voxel, and triple point parameters. Furthermore, the branches present are longer than those in wild-type animals. Finally, there is a reduction in the surface area to volume ratio of the entire NMJ. These characteristics can be see in Fig. 5a as these representative H304R/+ NMJs show fewer longer branches present. At three months of age the overall morphology of H304R/+ and wild-type NMJs were indistinguishable and the quantitative analysis was unable to identify any parameters with statistically significant differences (Fig. 5b, Table 1).

**Table 1:** Morphological changes in the NMJs are noticeable early in H304R/+ mice.

Time point	Genotype	*n*	*N*	% of NMJ Innervated	Branches	Junctions	End-point Voxels	Junction Voxels	Slab Voxels	Average Branch Length	Triple Points	Maximum Branch Length	Longest Shortest Path	Volume	Surface Area	SA/VOL
**1 month**	**wild-type**	**67**	**5**	95.4%	27.0 ± 11.9	15.3 ± 7.1	7.1 ± 2.8	29.2 ± 14.6	353 ± 141	6.1 ± 1.4	14.4 ± 5.8	19.6 ± 5.8	56.6 ± 15.8	687 ± 253	1524 ± 477	2.3 ± 0.3
	**H304R/**+	**88**	**5**	91.0%	23.3 ± 11.7	12.6 ± 7.1	7.9 ± 3.3	23.8 ± 13.9	312 ± 117	6.8 ± 2.1	11.7 ± 6.9	21.5 ± 7.9	60.6 ± 18.3	704 ± 247	1500 ± 486	2.2 ± 0.3
**3 month**	**wild-type**	**67**	**6**	93.9%	21.9 ± 9.1	11.6 ± 5.2	8.6 ± 3.1	21.8 ± 10.4	360 ± 112	7.6 ± 2.0	11.1 ± 4.9	22.3 ± 6.3	70.6 ± 20.3	1019 ± 400	1872 ± 561	1.9 ± 0.3
	**H304R/**+	**72**	**6**	97.2%	21.2 ± 11.9	11.0 ± 6.7	8.9 ± 3.9	21.5 ± 14.1	331 ± 146	8.1 ± 3.1	10.3 ± 6.2	24.0 ± 9.7	69.1 ± 21.7	896 ± 393	1738 ± 680	2.0 ± 0.3
**6 month**	**wild-type**	**98**	**5**	88.8%	19.3 ± 9.5	10.1 ± 5.4	7.6 ± 3.4	20.1 ± 13.5	335 ± 137	8.2 ± 2.9	9.5 ± 4.9	25.1 ± 8.9	68.9 ± 23.3	1278 ± 630	2021 ± 791	1.7 ± 0.3
	**H304R/**+	**82**	**5**	85.9%	18.3 ± 10.0	9.4 ± 5.7	8.1 ± 3.4	18.6 ± 13.3	313 ± 143	8.4 ± 2.9	8.9 ± 5.5	24.4 ± 9.4	69.9 ± 22.5	1430 ± 767	2060 ± 807	1.6 ± 0.3
**9 month**	**wild-type**	**63**	**5**	86.2%	22.0 ± 11.5	11.4 ± 6.6	9.1 ± 3.6	22.7 ± 17.6	363 ± 363	8.2 ± 3.2	10.8 ± 6.2	25.3 ± 9.0	74.7 ± 24.6	1052 ± 670	1925 ± 922	2.1 ± 0.5
	**H304R/**+	**65**	**5**	64.7%	18.2 ± 11.7	9.5 ± 7.0	7.6 ± 3.3	19.7 ± 17.8	301 ± 142	8.3 ± 3.1	9.08 ± 6.5	25.1 ± 9.5	68.2 ± 24.2	1159 ± 879	1813 ± 970	1.8 ± 0.6
**12 month**	**wild-type**	**118**	**6**	94.9%	22.8 ± 11.3	11.7 ± 6.4	9.7 ± 4.0	19.9 ± 12.8	388 ± 156	8.0 ± 2.5	11.2 ± 6.0	25.9 ± 9.5	78.5 24.4	1438 ± 719	2282 ± 907	1.7 ± 0.3
	**H304R/**+	**97**	**6**	73.9%	16.6 ± 8.9	8.5 ± 5.0	7.3 ± 3.3	21.7 ± 48.3	278 ± 144	7.9 ± 2.5	8.1 ± 4.7	23.2 ± 8.5	62.7 ± 26.4	1337 ± 742	1891 ± 895	1.5 ± 0.3

Structural changes in the AChRs occur early in the mutant mice but are statistically insignificant mid-way in the time course and later emerge at 9 months and become more pronounced at 12 months. The number of AChRs (n) and number of animals per genotype (N) are indicated on the table as well.

At six months of age we observed a small subset of H304R/+ NMJs that appeared less complex than the typical wild-type NMJ. However, these were only a fraction of the overall NMJs and the surface area/volume ratio was only parameter with a statistically significant difference between genotypes (Fig. 5c, Table 1). At the 9-month time point, there were a large percentage of H304R/+ NMJs with obvious morphological defects (Fig. 5d). Our analysis showed that there was a reduction in most of the quantitative parameters we measured and that reduction was statistically significant for four of the parameters in H304R/+ mice compared to wild-type littermate control mice (Table 1). We found the decrease in the percentage of NMJs that were innervated to be especially interesting as it may indicate that some form of degeneration may have occurred in these older NMJs.

The NMJs of 12-month old H304R/+ mice had severe defects from both qualitative and quantitative assessments. Many of the H304R/+ NMJs had a wider junction diameter with a notable reduction in the number of branches present (Fig. 5e). Our quantitative analysis of H304R/+ NMJs identified ten parameters with statistically significant decreases relative to age matched wild-type NMJs (Table 1). These differences are indicative of the gross abnormalities present in many of the H304R/+ NMJs. The percentage of NMJs that were innervated correctly was dramatically reduced in the H304R/+ NMJs at 12 months similar to the 9-month time point.

## Discussion

To gain understanding of the onset and progression of axonal CMT as well as the role of cytoplasmic dynein in cellular neuropathy, we have generated and initially characterized a knock-in mouse carrying the H304R mutation in the Dync1h1 cytoplasmic dynein heavy chain gene. This mutation corresponds exactly to the human H306R Dync1h1 mutation that results in CMT2O^10^. We examined both male and female H304R/+ mouse cohorts in a 12 month longitudinal assessment study to determine if the mutant Dync1h1 allele causes dominant locomotor deficits similar to those exhibited by individuals with CMT2; we also assessed whether H304R/+ male mice have neuromuscular pathologies.

H304R/+ male mice exhibit phenotypes that would be expected for a mouse model of CMT disease (Figs 2 and 3). The mice displayed muscle weakness, loss of motor coordination and atypical tail suspension reflexes compared to littermate controls. In addition, although our assay could not distinguish hind limb muscle strength directly, the progression of limb weakness from early onset “all limb” only to significant weakness in the forelimbs specifically at the later time points strongly suggests that the mouse disease pathology is following the progression of symptoms in humans from difficulty walking to progressive inability to use hands effectively.

Interestingly, we found differences in severity of phenotypes based on the sex of the animal, with female animals having weaker phenotypes overall compared to male counterparts when measuring grip strength and motor coordination but similar atypical tail suspension reflexes. We believe that our female H304R/+ mice exhibit milder CMT characteristics that will require more sensitive locomotor assays to distinguish. The initial study of human CMT Type 2 O characterized 8 males and 5 females in detail, with no obvious differences between the sexes being noted^10^. It is possible that our observed difference in expressivity of H304R/+ between males and females is due to specific sexual differences in the characteristics of mice; it will be extremely interesting to determine if the variation in expressivity occurs at the cellular level.

Based on the motor behavior phenotypes we observed, we decided to examine NMJ morphologies in male H304R/+ mice and saw significant differences in NMJ morphologies over time (Fig. 5, Table 1). We found that NMJs appeared under-developed at one-month of age, with fewer branches, and with those branches having significantly longer lengths. However, this phenotype disappeared at 3 months, as the NMJs were indistinguishable from control animals at that age. At later time points the NMJs lost complexity, and many times appeared to have a larger synapse diameter. One of the most interesting features of the H304R/+ phenotype was the apparent recovery of NMJ morphology in young adult mice followed by degeneration as the mice aged. The defects seen at the earliest time point seem to represent a problem in development of the NMJs, as less complexity was present. In contrast, the defects seen in the later time points suggests that there may be a separate mechanism in the older animals that is causing possible retraction or degeneration at the NMJ during aging. Future work will be needed to test those possibilities.

The severity of the tail suspension reflex, muscle weakness, and motor coordination defects in the H304R/+ mice appears less than that of the previously identified *Loa/*+, *Cra/*+, and *Swl/*+ dynein mutant mice^20,22–25^. Upon consideration, it may not be surprising that we see differences in phenotypes between different mouse models, as other studies have shown similar phenotypic differences depending on the exact genetic lesion present in other model organisms^18^. It appears likely that the function of cytoplasmic dynein is of such complexity that specific mutations may alter a small subset of its functions. In this case, the H304R/+ mouse is the only mouse known to date that harbors a DHC mutation that corresponds to the exact amino acid change that causes human disease. Although the defects seen in H304R/+ mice are less severe than those reported for *Loa/*+, *Cra/*+, and *Swl/*+ mice, that very difference may relate to the actual disease pathology in humans with lesions in the dynein gene. As such, we believe it will be an important model for understanding dynein function in complex organisms, especially with respect to CMT disease onset and progression.

## Methods

### Generation of DYNC1H1 H304R Knock-in Mice

An 8.04 kb region used to construct the targeting vector was subcloned from a positively identified C57BL/6 BAC clone (RP23:60K23). The BAC was subcloned into a 2.45 kb backbone vector (pSP72; Promega) containing an ampicillin selection cassette for retransformation of the construct prior to electroporation. The region was designed such that the long homology arm extended 5.3 kb 5′ to the point mutation (A to G) in exon 5 (Supp. Fig. 1). A pGK-gb2 FRT NeoR cassette was inserted into the gene in intron 5–6. The short homology arm extended 2.05 kb 3′ of the FRT-flanked Neo cassette. All primers used in the construction of the mouse are shown in Supplemental information.

The CMT2O mutation CAC to CGC (mouse aa304: His to Arg) within exon 5 was generated by 3-step PCR mutagenesis (Supp. Fig. 1). Four primers, PT1 – 3 and LUNI, were designed and used to amplify a ∼2.5 kb fragment that incorporated the mutation at the desired position. The point mutation was engineered into primers PT2 and PT3. The final PCR fragment carrying the point mutation (middle arm) was then used to replace the wild-type sequence using conventional sub cloning methods at an endogenous enzyme site, MfeI, and at a MluI site in the Neo cassette. The targeting vector was confirmed by restriction analysis after each modification step, and by sequencing using primers designed to read from the selection cassette into the 3′ end of the middle arm and the 5′ end of the SA. Sequencing showed the presence of the mutation and that no errors were introduced in the PCR amplified region.

The targeting vector was linearized and electroporated into C57BL/6 × 129/SvEv hybrid embryonic stem cells. After selection by G418 resistance, surviving colonies were expanded and screened for recombinant clones by PCR using primers LAN1 and A1 (Supp. Fig. 2). Positive clones had the presence of the point mutation confirmed by a second round of PCR screening using primers SQ1 and LUNI followed by sequencing of that PCR product. Integration into the correct genomic location was confirmed by southern blot analysis (Supp. Fig. 3). Three clones were confirmed as integrated into a parental chromosome at the targeted site and were then used for implantation.

Positive stem cells were microinjected into C57BL/6 blastocysts. The blastocysts were transferred to pseudopregnant foster mothers and chimeras were obtained. Those chimeric mice were crossed to mice constitutively expressing Flp recombinase to produce F1 heterozygous knock-in targeted, NeoR cassette deleted mice. PCR was used to confirm the deletion of the Neo cassette (Supp. Fig. 4) and sequencing of the four founder mice confirmed the presence of the point mutation.

The mouse colony was maintained by performing repeated backcrosses to B6129SF2/J mice (Jackson Laboratories stock number 101045). All animals were housed in microisolation cages with *ad libitum* access to food and water under controlled temperature (22 ± 2 °C) and humidity (50 ± 10%) and maintained on a 12-hour light/dark cycle. All mice were given a red housing tube and a wooden block for environmental enrichment. Pups were weaned on post-natal day 21. All animals were uniquely identified by permanent tail tattoo using Somark Tail Tattoo system (Somark Innovations). Tail snips were taken at the time of tattoo for PCR based genotyping utilizing the NDEL1 and NDEL2 primers to screen for the Neo cassette remnant linked to the point mutation.

Western blot analyses were performed to determine if the H304R mutation altered the amount or stability of dynein molecules in brain tissue. Brain tissue from wild-type and H304R/+ male mice was homogenized in 35 mM PIPES pH 6.96, 5 mM MgSO4, 1 mM EGTA, 0.5 mM EDTA supplemented with 1 mM dithiothreitol, 0.2 mM ATP, and a protease cocktail. A high-speed supernatant of each sample was generated by spinning the samples in an SW50.1 rotor for 30 minutes at 40,000 rpm. The samples were run on SDS-PAGE gels, blotted and probed using standard techniques, utilizing antibodies against dynein intermediate chain (clone 74.1, BioLegend) and glyceraldehyde-3-phosphate dehydrogenase (clone O411, Santa Cruz Biotechnology) as a loading control. Signal was detected using goat anti-mouse secondary antibody conjugated to horseradish peroxidase (Invitrogen) and the ECL Prime Western Blot Detection Reagent (Amersham). Signal was captured on X-ray film and quantified using NIH ImageJ software.

### Motor skills behavioral tests

All animal procedures were approved by the University of Central Florida IACUC committee and all experiments were performed in accordance with relevant guidelines and regulations. Mice were given motor skills behavior tests every two weeks beginning at 1 month of age. The researchers performing the behavioral tests were blinded to the genotype of the mice they were examining and the mice were only identifiable by tail tattoo codes. The tail suspension test was performed by gently lifting a mouse by its tail for 15 seconds. The test mouse was recorded from both ventral and lateral angles to fully capture the tail suspension reflex. The recorded tail suspension videos were then analyzed and each test was scored into one of two categories: typical or atypical response. Mice exhibiting a typical response had their hind limbs splayed apart from each other and held away from the body. In an atypical response, mice hind limbs were clenched together and held either away from or near the body. The categorical tail suspension data for mice of different genotypes were binned in three-month intervals and statistically compared using the Fisher’s exact test (two-tailed distribution).

The accelerating rotarod test utilized an instrument (IITC Life Science, model# 755) programmed to start at 5 RPM, ramp to 40 RPM over 60 seconds, and then maintain 40 RPM speed for a total time of three minutes. The elapsed time until the mouse fell off the rotating rod was automatically collected with the Series 8 software provided with the instrument. Each mouse performed three successive runs and these 3 readings were averaged for each test day. Each test mouse’s averaged rotarod reading from test days was averaged again for 3-month bins. The wild-type and H304R/+ binned rotarod datasets were statistically compared for 3-month bin using the two-tailed Student’s t-test.

The limb muscular strength of mice was measured with a digital grip strength instrument (BIOSEB Research Instruments, model# BIO-GS3). Each test mouse was placed on the steel grid attached to the instrument and the grip strength reading was collected as per the manufacturer’s protocol. The manufacturer’s RSIC software recorded the maximum force (in grams) exerted by the mouse on the steel grid. The grip strength in the front two limbs and all four limbs were measured for each test mouse. Mice could not grip the instrument solely with hind limbs, preventing the direct assessment of hind limb strength. All the grip strength measurements were taken in quadruplicates and the four readings were averaged on each test day. Each test mouse’s averaged grip data from test days was averaged for 3-month bins. The wild-type and H304R/+ binned grip datasets were statistically compared using the two-tailed Student’s t-test.

### Tissue Preparation

Animals were anesthetized via intraperitoneal injection with SomnaSol ™ (7.5 mg/kg, Henry Schein). Cardiac perfusion was performed using 25 ml of 0.9% saline solution and then 25 ml of 4% paraformaldehyde. Gastrocnemius muscles were carefully dissected from animals and incubated in 4% paraformaldehyde for up to two hours. The muscles were transferred into 30% sucrose for cryopreservation overnight at 4 °C. Muscles were embedded in O.C.T. compound (Sakura Finetek) in plastic cryomolds (Ted Pella Inc.) and stored at −80 °C until ready for sectioning. 20 µm sections were obtained from muscles using a cryostat (Leica Biosystems CM1850) and were attached to Superfrost Plus glass slides (Thermo Fisher Scientific). Slides were immersed in acetone for 20 min at room temperature and then stored at −20 °C until used for imaging.

### Immunohistochemistry and Image Analyses

Cryosections were washed twice with PBS for 10 min to remove the O.C.T. compound. The edges of the sections were marked with a hydrophobic ImmEdge Pen (Vector Labs) to restrict the staining solution. Sections were permeabilized for 10 min with 0.5% Triton X100 and blocked for up to three hours in a block solution of 5% bovine serum albumin, 5% goat serum, and 0.1% TritonX100 in PBS at room temperature. Staining was done in a humidified chamber with neurofilament specific antibodies (2H3 (Developmental Studies Hybridoma Bank) or NF-H (Millipore Sigma) at 4 °C overnight. The sections were then washed three times with block solution. All subsequent steps were carried out at room temperature. Block solution containing a mixture of appropriate AlexaFluor-546 secondary antibodies (Life Technologies) and AlexaFluor-488 alpha-bungarotoxin (Life Technologies) was added for 60 min to allow visualization of both neurofilaments and acetylcholine receptors (AChR). The sections were washed twice with block solution and twice with PBS. The sections were mounted with Prolong Gold antifade reagent containing DAPI (Life Technologies) to stain nuclei. Primary antibodies against alpha actinin (Abcam) and appropriate secondary antibodies were used similarly to examine sarcomere organization of gastrocnemius muscles.

Images were captured with the 63× oil immersion objective of a Zeiss LSM 710 Confocal microscope using the Zen software. Each resulting slice of the image had a depth of 8 bits and dimensions of 134.95 µm × 134.95 µm × 1 µm. The images were acquired at a resolution of 3.794 pixels per µm. The following fluorophores were utilized in imaging at their respective excitation (Ex) and emission (Em) wavelengths: DAPI Ex-358 nm, Em-461 nm, Alexa Fluor 488 Ex- 495 nm, Em-519nm, Alexa Fluor 546 Ex- 532 nm, Em-573 nm or DyLight 405 Ex- 400 nm, Em-420 nm.

Stacks obtained from confocal imaging were processed further using ImageJ software for detailed analysis of NMJ innervation patterns and AChR architecture. The respective confocal channels corresponding to neurofilament and AChR staining were merged and then analyzed using the “*3D Viewer”* plug-in to determine innervation at the NMJs^27^. We classified NMJs as innervated if more than half of the receptor volume displayed a fluorescent signal for neurofilament.

To measure the volume and surface area of the NMJs, images were prepared by using the process *‘smooth’* to reduce AChR signal noise and by applying a binary mask to portray dark images against a white background. The threshold was set to exclude objects that were outside the region of interest. Volume and surface area were then calculated using the “*3D object counter”* plug in. We utilized the “*Skeletonize 2D/3D”* and “*Analyze Skeleton 2D/3D”* plug-ins to generate a three dimensional single line version of the NMJ and evaluate other parameters of the resulting skeletonized NMJ^28^. Detailed definitions of the parameters are provided in the Supplemental Information section. The Student’s *t*-test or Chi-square analysis (for differences in innervation) were used to determine the statistical significance of data comparisons between different genotypes.

The datasets generated during and/or analyzed during the current study are available from the corresponding author on reasonable request.

## Electronic supplementary material

Supplemental Information
