# Supplementary material for: A novel mouse model carrying a human cytoplasmic dynein mutation shows motor behavior deficits consistent with Charcot-Marie-Tooth type 2O disease
Source: Sci Rep. 2018 Jan 29;8:1739. doi: 10.1038/s41598-018-20081-1 (PMC5789002; doi:10.1038/s41598-018-20081-1)
Supplement: Supplementary file 1 — Supplemental Information [file 41598_2018_20081_MOESM1_ESM.pdf]

Supplemental Information For:

A novel mouse model carrying a human cytoplasmic dynein mutation shows motor behavior deficits consistent with Charcot-Marie-Tooth type 2O disease

Thywill T. Sabblah, Swaran Nandini, Aaron P. Ledray, Julio Pasos, Jami L. Conley Calderon, Rachal Love, Linda E. King, and Stephen J. King

Burnett School of Biomedical Sciences
College of Medicine
University of Central Florida
Orlando, FL 32827, USA

Supplemental Information:

Sequences for primers used in the construction of the H304R/+ mouse as well as supporting figures characterizing the molecular genetics of the mouse are provided here.

Primers for mutagenesis:
PT1: 5’ AGACGACGACTTGTGTGGCAATTG
PT2: 5’ GCGCTTCAGGATGTCCAGAGTCAGG
PT3: 5’ CTGGACATCCTGAAGCGCGGCAAACGTTTCCATGCCACTG
LUNI: 5’ GCATCGCCTTCTATCGCCTTCTTG

Primers for screening of recombinant clones:
A1: 5’ AACATCTCATTGGCATTCTTGGCC
LAN1: 5’ CCAGAGGCCACTTGTGTAGC

Primers for PCR screening of point mutation:
SQ1: 5’ACTTGAGATTTACCAGGCGATGTCATC
LUNI: 5’ GCATCGCCTTCTATCGCCTTCTTG

Primers for genotype screening of mice:
NDEL1: 5’ GATGACCGACTAGGCCATTAAAG
NDEL2: 5’ GGGTGGTAGGATGGGGAAGTAGAG

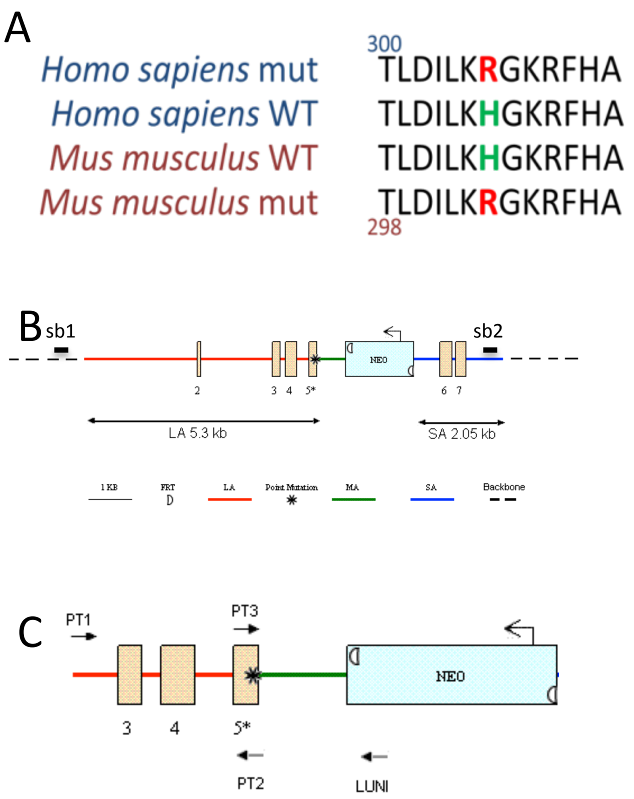

Sup Fig 1 A) Comparison of dynein heavy chain sequences from human and mice near the H306R human mutation. B and C): Schematic models of targeting vector and adjacent genomic DNA. B) Orientation of the targeting vector elements. Positions of sb1 and sb2 probes used for Southern blotting are given. C) Expanded view illustrating the generation of the point mutation by PCR. PT2 and PT3 had the mutation incorporated into the primer sequence.

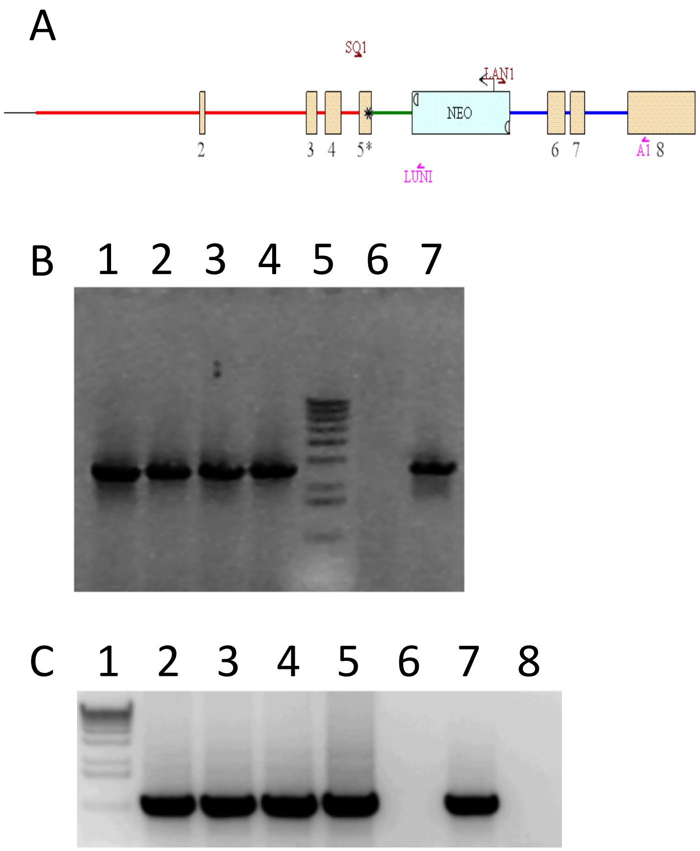

Sup Fig 2 Confirmation of expanded clones by PCR. A) schematic of the targeting vector, selected primer locations, and adjacent genomic DNA. B) Lanes 1-4: DNA from selected clones; 5: DNA ladder; 6: wild type DNA, 7:vector DNA. Primers A1 and LAN1 were used to amplify DNA by PCR. The clones in lanes 1-4 amplify the predicted target 2.3 kb fragment that contains a portion of the Neo cassette. C) Lane 1 DNA ladder; 2-5: DNA from selected clones; 6: wild type DNA, 7: vector DNA. 8: no DNA. Primers SQ1 and LUNI were used to amplify DNA by PCR. The clones in lanes 2-5 amplify the predicted target 1.03 kb fragment that contains the point mutation. This product was later utilized for sequencing.

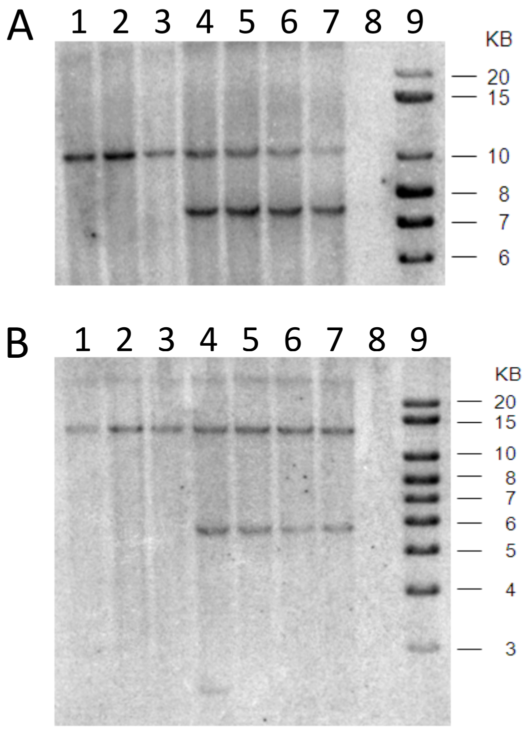

Sup Fig 3

Genomic targeted integration analyzed by Southern blot analysis. Lanes 1: 129 parent, 2: B6 parent, 3:hybrid, 4-7: individual clones, 8: no DNA, 9: DNA ladder. A) The blot was probed with the sb1 probe located external to the vector (Sup Fig. 1). This probe is predicted to generate a band of 10140 bp in parental strains and a 7447bp band upon correct integration. B) The blot was probed with the sb2 probe located in intron 7-8. This probe should generate a 12923 bp band in parental strains and 5661 bp band upon successful targeted integration. The clones in lanes 5-7 were used for implantation.

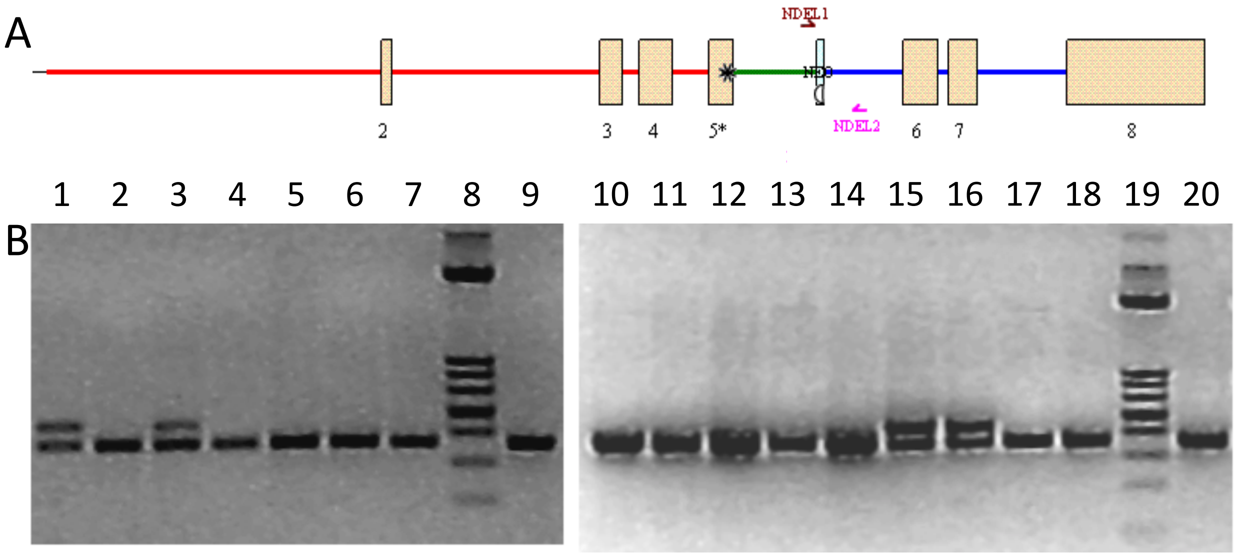

Sup Fig 4 PCR screening of the removal of the NEO cassette after crossing to C57BL/6 FLP mice. A) Schematic showing the location of the primers NDEL1 and NDEL2 used for screening. B) Lanes 1-7, 10-18: PCR screens from individual mice; Lanes 8 & 19: DNA ladder; Lanes 9 & 20: PCR screens from wild type mice. All mice product a PCR product of 368 bp. A 63 bp remnant of the NEO cassette causes the production on a second PCR product of 431 bp after successful removal of the NEO cassette. The mice identified in lanes 1, 3, 15, and 16 showed the presence of the NEO cassette remnant and became the founder mice of the colony.

	Time point	Wild-type (*n*)	H304R/+ (*n*)	*p*-value
**Male mice**	WT vs H304R/+ 3 months	34	25	0.0745
	WT vs H304R/+ 6 months	46	34	0.0779
	WT vs H304R/+ 9 months	29	27	0.0149
	WT vs H304R/+ 12 months	40	42	0.0002
**Female mice**	WT vs H304R/+ 3 months	9	8	0.6928
	WT vs H304R/+ 6 months	35	21	0.3569
	WT vs H304R/+ 9 months	54	37	0.0363
	WT vs H304R/+ 12 months	56	44	0.0386

Supplemental Table 1. Tail suspension test - Number of animals (n), and Fisher's exact test (two-tailed distribution) *p*-values for the wild-type and H304R/+ (heterozygous) mice

Test	Time point	Wild-type (mean)	H304R/+ (mean)	Wild-type (*n*)	H304R/+ (*n*)	*p*-value
All limbs grip strength	4-13 weeks	212.5 ± 23.2	190.4 ± 26.8	50	43	0.0001
	14-26 weeks	225.1 ± 30.8	201.0 ± 29.9	62	58	0.0001
	27-39 weeks	230.6 ± 37.9	206.8 ± 33.4	41	42	0.0033
	40-52 weeks	244.5 ± 32.1	225.3 ± 33.5	40	42	0.0096
Front limbs grip strength	4-13 weeks	99.2 ± 12.9	95.6 ± 17.4	50	43	0.2654
	14-26 weeks	88.8 ± 14.3	83.8 ± 15.0	62	58	0.0667
	27-39 weeks	89.8 ± 12.9	82.1 ± 11.1	41	42	0.0045
	40-52 weeks	92.0 ± 11.9	86.1 ± 13.4	40	42	0.0362
Rotarod	4-13 weeks	78.8 ± 40.9	55.2 ± 30.4	51	43	0.0019
	14-26 weeks	95.5 ± 49.1	73.0 ± 42.4	48	41	0.0223
	27-39 weeks	101.9 ± 52.9	74.6 ± 44.8	28	33	0.0359
	40-52 weeks	100.7 ± 48.6	82.4 ± 45.7	20	25	0.2035

Supplemental Table 2. Male behavior mice - Mean values (± s.d.), number of animals (n), and the t-test (two-tailed distribution) *p*-values for the male wild-type and H304R/+ (heterozygous) mice

Test	Time point	Wild-type (mean)	H304R/+ (mean)	Wild-type (*n*)	H304R/+ (*n*)	*p*-value
All limbs grip strength	4-13 weeks	175.5 ± 24.0	171.9 ± 22.0	51	35	0.4820
	14-26 weeks	192.9 ± 15.9	185.7 ± 16.9	56	41	0.0324
	27-39 weeks	209.8 ± 18.8	199.8 ± 16.5	55	41	0.0068
	40-52 weeks	222.2 ± 22.2	216.4 ± 20.0	54	41	0.1843
Front limbs grip strength	4-13 weeks	91.0 ± 12.5	89.1 ± 12.2	51	35	0.4801
	14-26 weeks	81.5 ± 9.8	81.8 ± 11.0	56	41	0.8959
	27-39 weeks	81.3 ± 11.8	79.8 ± 11.1	55	41	0.5148
	40-52 weeks	85.9 ± 13.4	85.4 ± 13.1	54	41	0.8681
Rotarod	4-13 weeks	103.6 ± 47.6	82.7 ± 34.3	51	35	0.0205
	14-26 weeks	113.7 ± 54.9	104.9 ± 45.2	51	35	0.4210
	27-39 weeks	106.8 ± 61.4	105.9 ± 54.0	51	35	0.9386
	40-52 weeks	98.8 ± 62.6	87.7 ± 51.3	50	35	0.3711

Supplemental Table 3. Female behavior mice - Mean values (± s.d.), number of animals (n), and the t-test (two-tailed distribution) *p*-values for the female wild-type and H304R/+ (heterozygous) mice

Definitions of Parameters Used in Characterizing Neuromuscular Junction Architecture

**Branches**	The number of elongated segments in the skeletonized NMJ that link a junction point to the nearest junction or end point.
**Junctions**	The number of vertices in the skeletonized NMJ that connect two or more branches.
**Triple points**	The number of vertices in the skeletonized NMJ that connect three branches.
**Voxels**	The three-dimensional building blocks (“pixels”) in the 3D skeletonized NMJ.
**Endpoint voxels**	The number of voxels with only one neighbor voxel.
**Junction voxels**	The number of voxels with more than two neighbor voxels.
**Slab voxels**	The number of voxels with two neighbor voxels.
**Average branch length**	The mean branch length of all branches in the skeletonized NMJ.
**Max branch length**	The length of the longest branch in the skeletonized NMJ.
**Longest shortest path**	The shortest path distances between all possible endpoint pairs in the skeletonized NMJ are determined. The longest of these shortest path distances is then identified.
**SA/Vol**	The surface area to volume ratio of the NMJ.
